# Supplementary material for: Predictive neural representations of naturalistic dynamic input
Source: Nat Commun. 2023 Jun 29;14:3858. doi: 10.1038/s41467-023-39355-y (PMC10310743; doi:10.1038/s41467-023-39355-y)
Supplement: Supplementary file 1 — Supplementary info [file 41467_2023_39355_MOESM1_ESM.pdf]

# Predictive neural representations of naturalistic dynamic input

Ingmar E.J. de Vries*[1,2], Moritz F. Wurm[1]

[1] Centre for Mind/Brain Sciences (CIMeC), University of Trento, 38068 Rovereto, Italy.
[2] Donders Institute, Radboud University, 6525 EN Nijmegen, The Netherlands

* i.e.j.de.vries@gmail.com

## Supplementary Figures

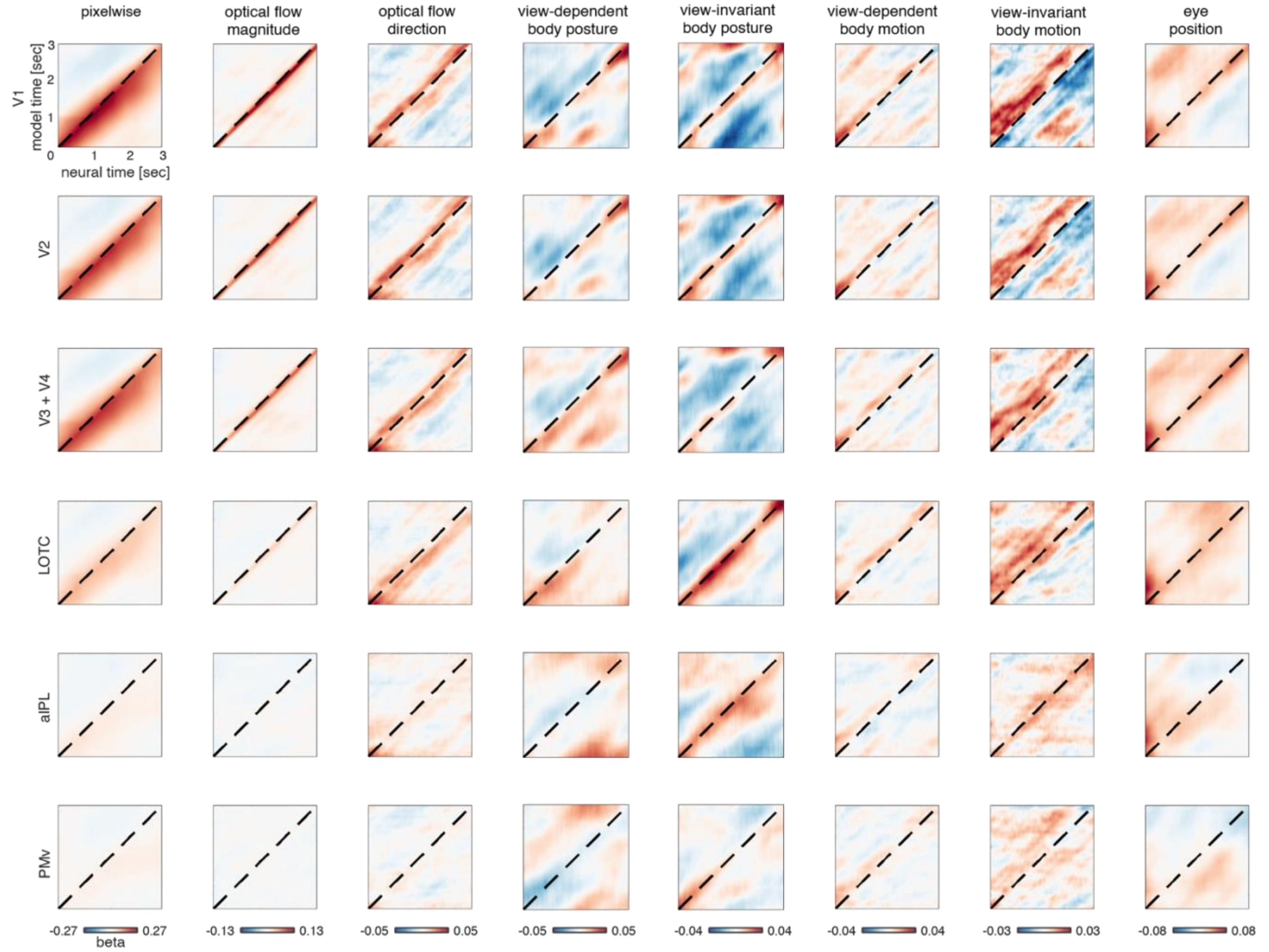


**Supplementary Figure 1. Neural-time by model-time two-dimensional dRSA.** Dynamic RSA regression weights, illustrated as 2-dimensional model-time by neural-time dRSA matrix, where stronger weights in the lower right triangle indicate that the neural representation is lagging behind a model representation, while stronger weights in the upper left triangle indicate that the neural representation is preceding a model representation (i.e., a predictive neural representation). Different stimulus-feature models are plotted in columns, while ROIs are plotted in rows. The diagonal dashed line indicates zero lag between the neural and model RDM. Note that the scale illustrated below each column reflects the scale for that whole column.

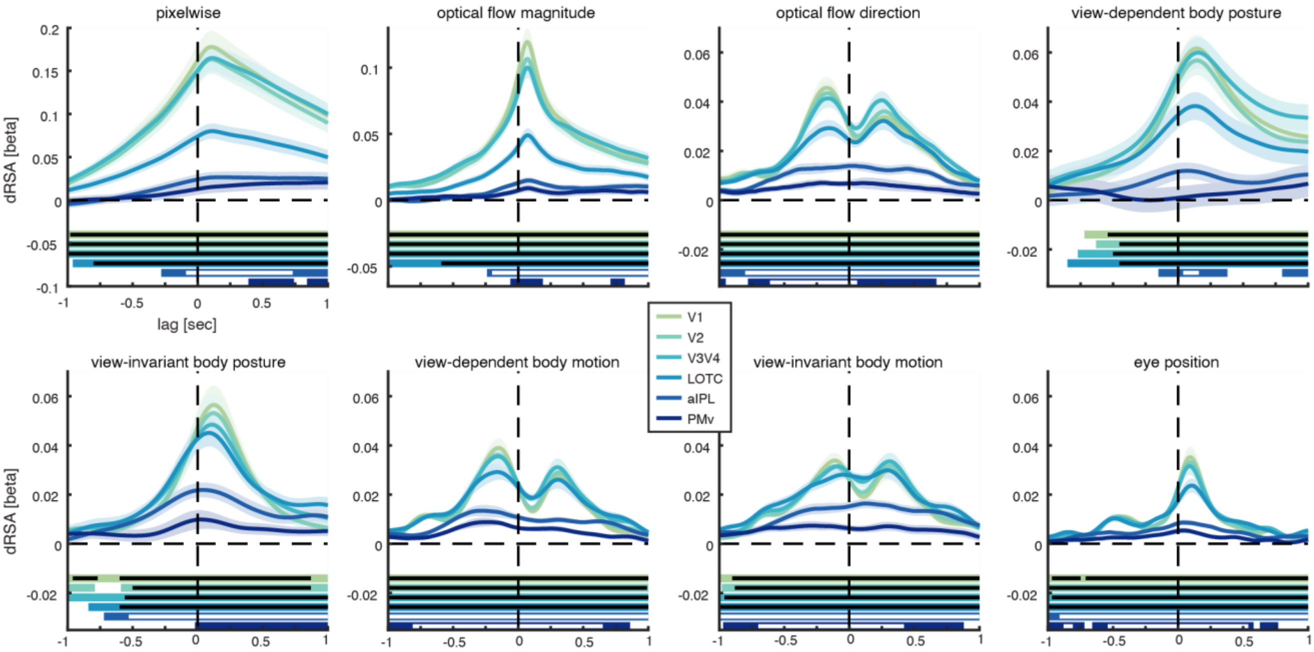


**Supplementary Figure 2. ROI-based dRSA results with correlation instead of PCR as similarity measure.** Exact same analysis as presented in Figure 2 with the same conventions for colors, subplots, significant intervals, etc., but with correlation coefficient instead of regression weights as similarity measure. That is, the neural RDM is correlated with each model RDM separately.

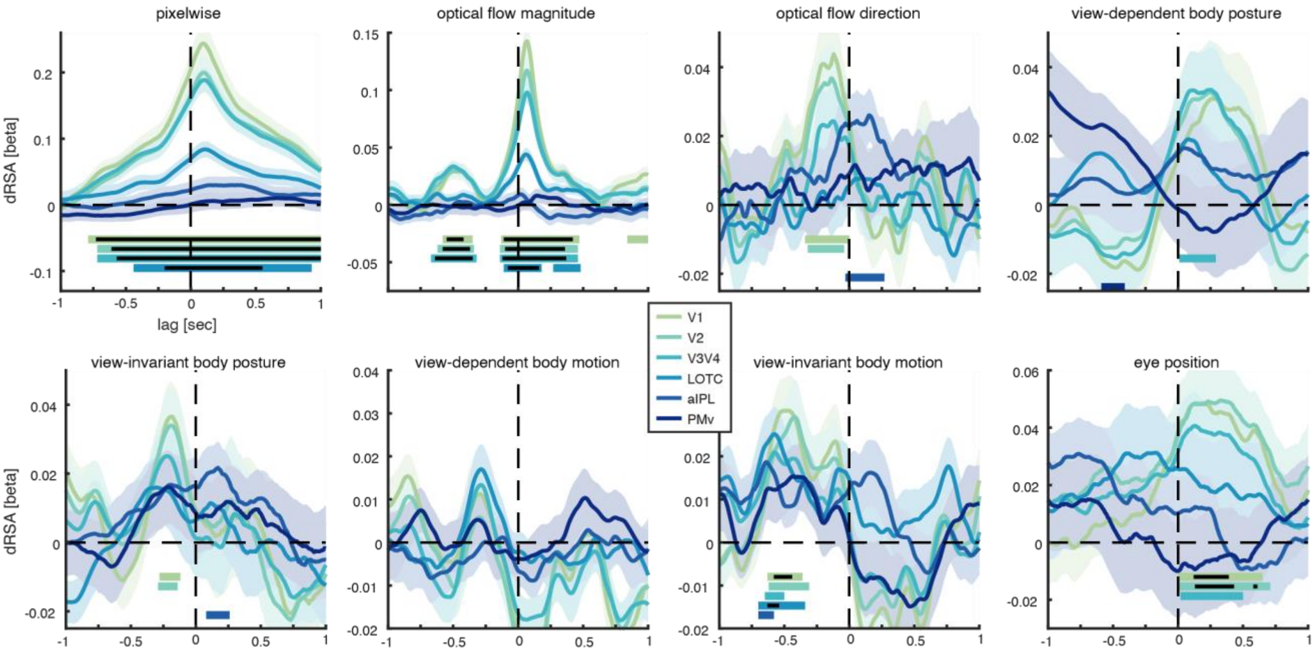


**Supplementary Figure 3. ROI-based dRSA results without temporal subsampling step.** Exact same analysis as presented in Figure 2 with the same conventions for colors, subplots, significant intervals, etc., but without the temporal subsampling and realignment step. That is, the dRSA analysis is performed directly on the original 5-sec stimuli.

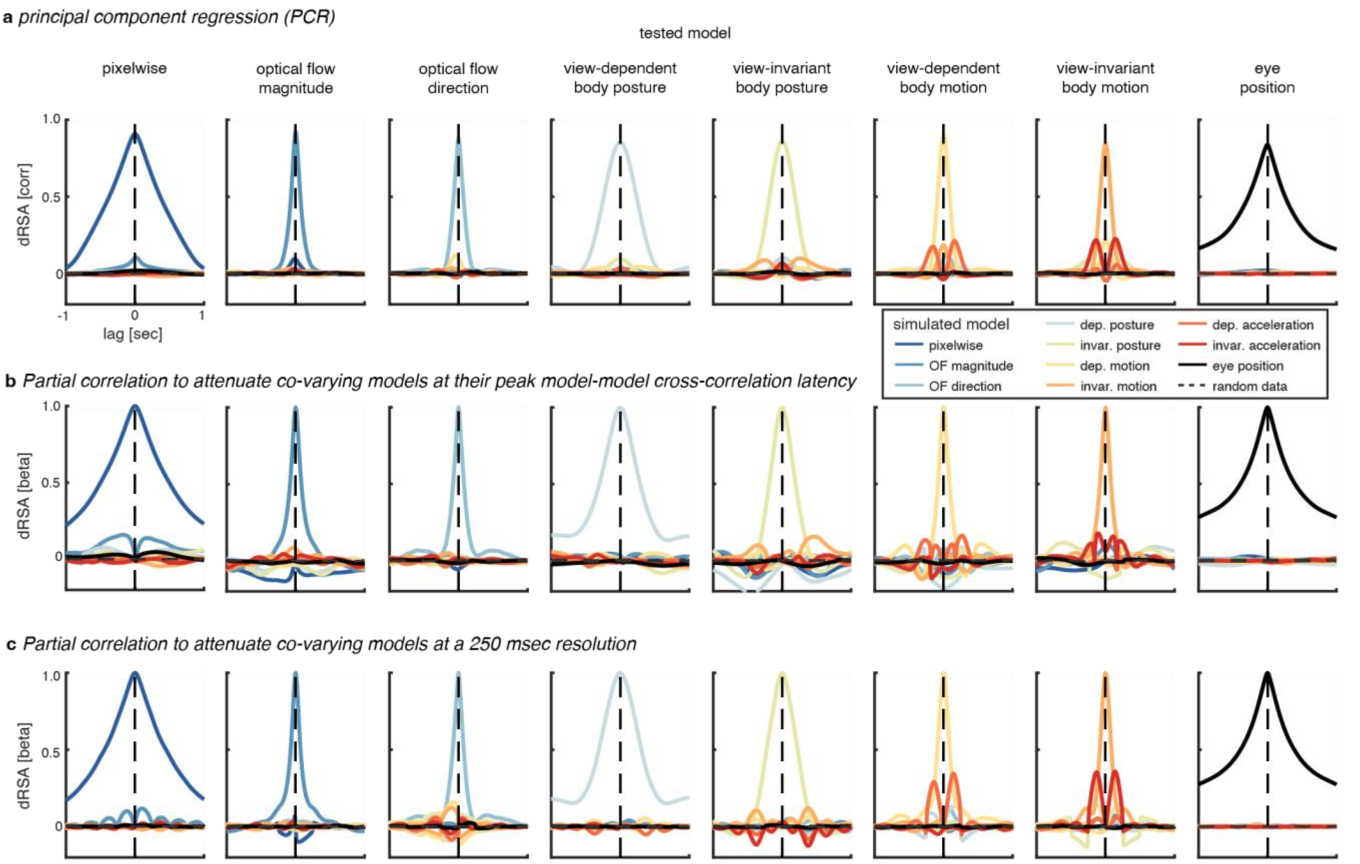


**Supplementary Figure 4. Comparison of similarity measures on simulated data. a** Regression weight as similarity measure for dRSA (as in Fig. 8b). Colors indicate separate neural simulations of individual model RDMs. Columns indicate which model was tested. **b** Partial correlation as similarity measure for dRSA which included besides the model of interest all other models at the latency at which each individual model correlated strongest with the model of interest. Specifically, first peak latencies of the cross-model correlations were extracted from Figure 8a, after which a partial correlation that included the model of interest at a certain timepoint $t_x$, as well as all other models at their respective peak latencies in the cross-correlation with the model of interest. **c** Partial correlation as similarity measure for dRSA that included besides the model of interest all other models after downsampling (250 msec). Importantly, these results indicate that PCR is most effective at extracting only the model of interest from the simulated neural RDM, while attenuating the effect of co-varying models.

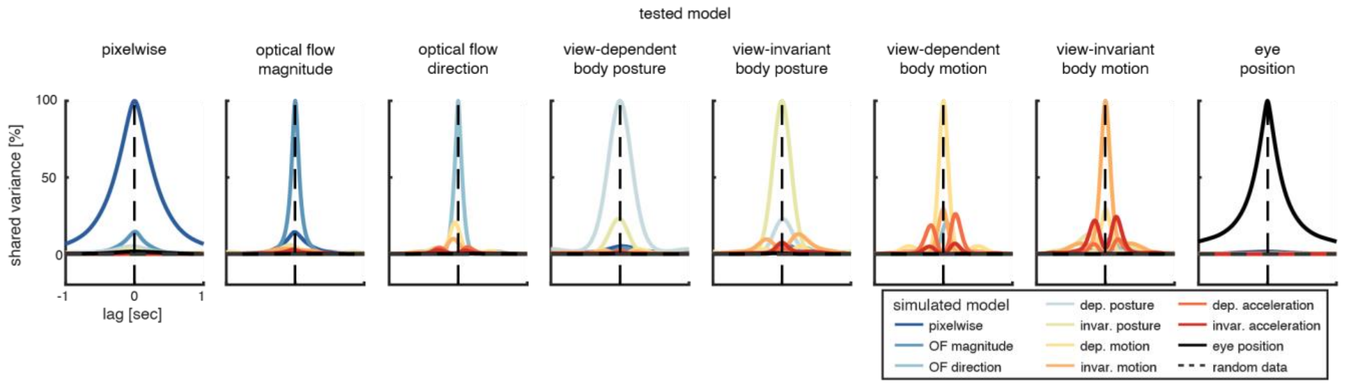


**Supplementary Figure 5. Shared variance between models.** Exact same data as figure 8a (i.e., cross-model correlations) but squared and multiplied by 100%.

# Supplementary Discussion

## No effect of stimulus familiarization throughout experiment

Given that the 14 unique videos were each repeated ~36.4 times, one might expect stimulus familiarization throughout the experiment to improve prediction, for instance by lengthening the forecast window, or by strengthening the predictive representation. To test this, we ran the dRSA analysis on the first and last 1/3 of the trials (Supplementary Fig. 6). Most importantly, we did not find significant differences in the predictive representations. Interestingly, we did observe a reduction in the lagged representation of view-dependent body posture in the later trials, and a trend for a reduction in the lagged representation of optical flow direction in later trials, both of which would be in line with a predictive processing account: i.e., lagged representations would only reflect prediction errors and would thus be expected to reduce with more stimulus knowledge. However, only the effect in view-dependent body motion was significant, and only at our more lenient statistical threshold ($p < 0.05$), thus making this interpretation highly speculative.

One explanation for not finding a robust difference is that it was very difficult for subjects to learn the ballet sequences, as the 14 different combinations of 4 ballet figures, selected from only 5 possible figures, made it hard to predict the next figure at any given time. E.g., if the first figure was a bow, the second could still be an arabesque, passé or jump, and was thus not predictable (see Table 1). This was confirmed by the fact that when anecdotally asked afterwards, subjects did not subjectively experience that they could predict the next ballet figure in the sequence. We therefore believe that the observed predictions are mainly enabled by prior knowledge on biological motion (and gravitation) that is already available to the subjects, rather than new stimulus knowledge/familiarization acquired throughout the experiment. To test the effect of stimulus familiarization (or prior stimulus knowledge) properly, it would be interesting in future research to e.g., compare expert observers to novices, or compare observers pre- and post-training of completely new stimulus material (i.e., not biological motion). Last, note that even if dRSA values are generally lower (due to lower SNR when averaging over less trials), the results are relatively comparable to the main results (Fig. 2), indicating that dRSA works also with less data. In other words, if one so wishes, in future dRSA research it would be possible to use more unique stimuli (i.e., larger RDMs) with fewer repetitions.

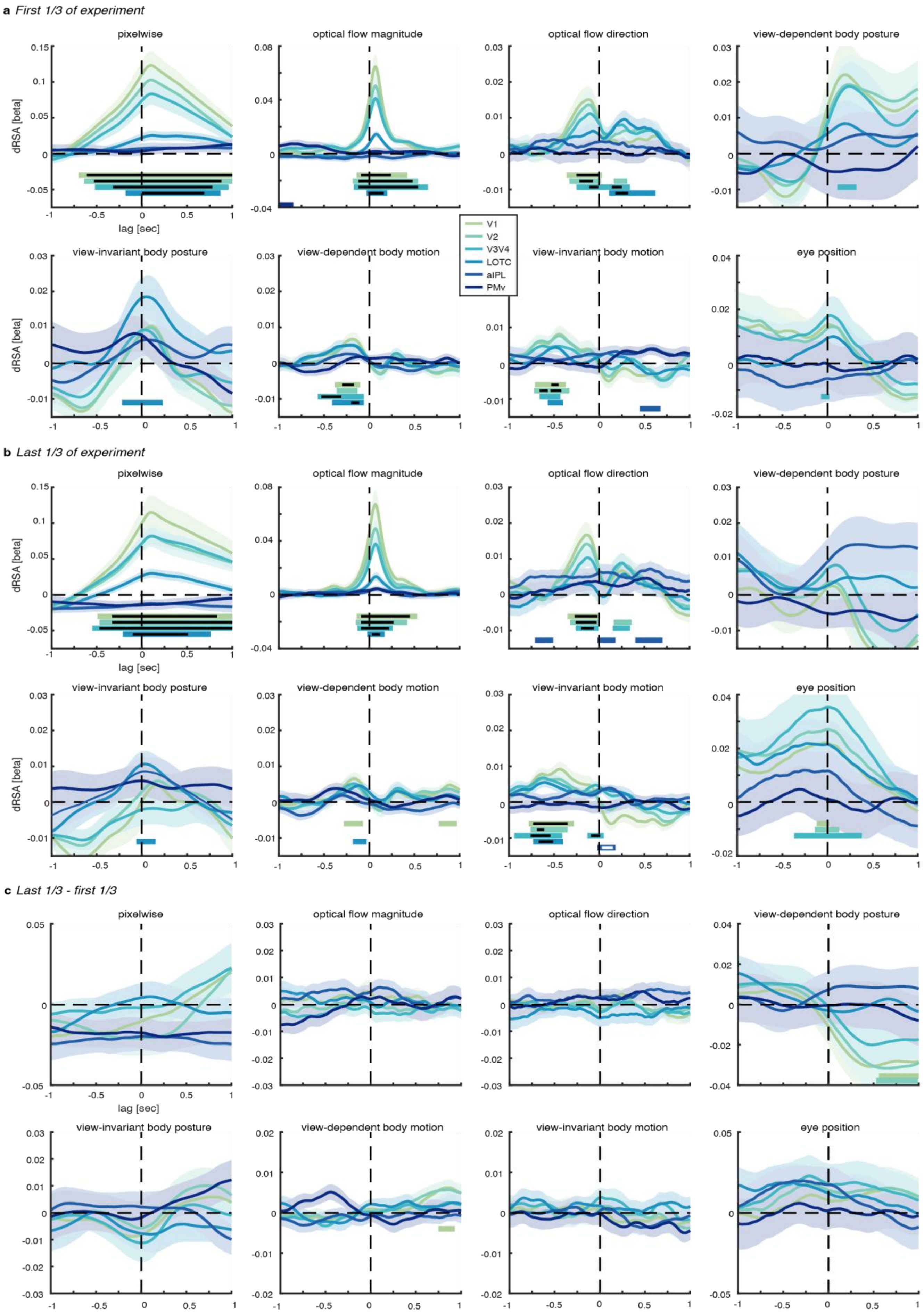


**Supplementary Figure 6. ROI-based dRSA results on first or last 1/3 of trials.** Exact same analysis as presented in Figure 2 with the same conventions for colors, subplots, significant intervals, etc., but on only first (**a**) or last (**b**) 1/3 of trials. **c** The difference between results in **a** and **b**.
