# Supplementary material for: Predictive neural representations of naturalistic dynamic input
Source: Nat Commun. 2023 Jun 29;14:3858. doi: 10.1038/s41467-023-39355-y (PMC10310743; doi:10.1038/s41467-023-39355-y)
Supplement: Supplementary file 3 — Reporting Summary [file 41467_2023_39355_MOESM3_ESM.pdf]

nature portfolio

Corresponding author(s): Ingmar E.J. de Vries

Last updated by author(s): May 17, 2023

Reporting Summary

Nature Portfolio wishes to improve the reproducibility of the work that we publish. This form provides structure for consistency and transparency in reporting. For further information on Nature Portfolio policies, see our Editorial Policies and the Editorial Policy Checklist.

Statistics

For all statistical analyses, confirm that the following items are present in the figure legend, table legend, main text, or Methods section.

n/a	Confirmed	
☐	☒	The exact sample size (*n*) for each experimental group/condition, given as a discrete number and unit of measurement
☐	☒	A statement on whether measurements were taken from distinct samples or whether the same sample was measured repeatedly
☐	☒	The statistical test(s) used AND whether they are one- or two-sided<br>*Only common tests should be described solely by name; describe more complex techniques in the Methods section.*
☐	☒	A description of all covariates tested
☐	☒	A description of any assumptions or corrections, such as tests of normality and adjustment for multiple comparisons
☐	☒	A full description of the statistical parameters including central tendency (e.g. means) or other basic estimates (e.g. regression coefficient) AND variation (e.g. standard deviation) or associated estimates of uncertainty (e.g. confidence intervals)
☐	☒	For null hypothesis testing, the test statistic (e.g. *F*, *t*, *r*) with confidence intervals, effect sizes, degrees of freedom and *P* value noted<br>*Give P values as exact values whenever suitable.*
☒	☐	For Bayesian analysis, information on the choice of priors and Markov chain Monte Carlo settings
☒	☐	For hierarchical and complex designs, identification of the appropriate level for tests and full reporting of outcomes
☒	☐	Estimates of effect sizes (e.g. Cohen's *d*, Pearson's *r*), indicating how they were calculated

Our web collection on statistics for biologists contains articles on many of the points above.

Software and code

Policy information about availability of computer code

Data collection	Custom MATLAB (version 2012b; MathWorks) code and the MATLAB-based Psychophysics Toolbox (version 3) was used for data collection. The experimental script is freely available at https://github.com/Ingmar-de-Vries/DynamicPredictions.
Data analysis	Custom MATLAB (version 2020a; MathWorks) code and MATLAB-based toolboxes Brainstorm (version 3), Fieldtrip (version 20191113) and CoSMoMVPA (version 1.1.0) were used for data analysis. All custom code is freely available at https://github.com/Ingmar-de-Vries/DynamicPredictions. Additionally, CAT12 (version 2170) as implemented in Brainstorm was used for MRI segmentation, Neuromag's MaxFilter (version 2.2) was used for maxfiltering the MEG signals, and the Farneback algorithm as implemented in MATLAB was used for computing optical flow vectors.

For manuscripts utilizing custom algorithms or software that are central to the research but not yet described in published literature, software must be made available to editors and reviewers. We strongly encourage code deposition in a community repository (e.g. GitHub). See the Nature Portfolio guidelines for submitting code & software for further information.

Data

Policy information about availability of data

All manuscripts must include a data availability statement. This statement should provide the following information, where applicable:

- Accession codes, unique identifiers, or web links for publicly available datasets
- A description of any restrictions on data availability
- For clinical datasets or third party data, please ensure that the statement adheres to our policy

The raw and processed MEG data are available under restricted access, as the authors are still using this MEG dataset for a successive project. Access can be obtained by request to the corresponding author. As soon as the authors are finished with the dataset, it will be made publicly available. However, note that a complete dataset of a single subject is already available at OSF such that the analysis pipeline can be tested (DOI: 10.17605/OSF.IO/ZK42F). Source data are provided with this paper, and available on the OSF at DOI 10.17605/OSF.IO/ZK42F. Last, this study made use of two open source atlases implemented in Brainstorm: The Human Connectome Project (HCP) atlas and the Schaefer atlas.

Human research participants

Policy information about studies involving human research participants and Sex and Gender in Research.

Reporting on sex and gender	13 out of 22 participants identified themselves as female sex. Gender information has not been collected otherwise. No sex- or gender-based analyses were performed.
Population characteristics	Healthy human subjects aged 30 (std 7)
Recruitment	Participants were recruited using a social media (Facebook) account from university that has the specific purpose of subject recruitment, and personal communication. Note that this recruitment procedure resulted in most subjects being university students and young researchers (BSc, MSc, PhD or postdoc), with an interest in research, which may therefore reduce generalizability of the findings. We foresee no other biases in the recruitment.
Ethics oversight	Ethical Committee of the University of Trento, Italy

Note that full information on the approval of the study protocol must also be provided in the manuscript.

Field-specific reporting

Please select the one below that is the best fit for your research. If you are not sure, read the appropriate sections before making your selection.

☒ Life sciences ☐ Behavioural & social sciences ☐ Ecological, evolutionary & environmental sciences

For a reference copy of the document with all sections, see nature.com/documents/nr-reporting-summary-flat.pdf

Life sciences study design

All studies must disclose on these points even when the disclosure is negative.

Sample size	No sample size calculation was performed, but a minimum sample size was chosen based on seminal MEG-based RSA studies (e.g., Cichy et al. (2014) Nat. Neurosci. - 16 subjects, Cichy et al. (2016) Sci. Rep. - 15 subjects, Kietzmann et al. (2019) PNAS - 15 subjects).
Data exclusions	No participant was excluded. Noisy sensors were interpolated, noisy trials were rejected (see main text for details).
Replication	The results presented in this article are based on a single experiment. The experiment was not replicated.
Randomization	This was a within-subject design, meaning that all subjects performed all conditions in random order: i.e., the 14 stimulus sequences used for dynamic RSA were presented in a randomized order, with the only constraint that the exact same sequence was never directly repeated (i.e., there was always at least one different sequence in between).
Blinding	There were no group allocations; therefore, blinding was not necessary/applicable

Reporting for specific materials, systems and methods

We require information from authors about some types of materials, experimental systems and methods used in many studies. Here, indicate whether each material, system or method listed is relevant to your study. If you are not sure if a list item applies to your research, read the appropriate section before selecting a response.

Materials & experimental systems

n/a	Involved in the study
☒	☐ Antibodies
☒	☐ Eukaryotic cell lines
☒	☐ Palaeontology and archaeology
☒	☐ Animals and other organisms
☒	☐ Clinical data
☒	☐ Dual use research of concern

Methods

n/a	Involved in the study
☒	☐ ChIP-seq
☒	☐ Flow cytometry
☒	☐ MRI-based neuroimaging
